# Secondary Displacement of Forearm Fractures in Children: When to Anticipate Remodeling and When to Intervene?

**DOI:** 10.3390/children13010098

**Published:** 2026-01-09

**Authors:** Kasper C. Roth, Linde Musters, Leon W. Diederix, Pim Edomskis, Christiaan J. A. van Bergen, Denise Eygendaal, Joost W. Colaris

**Affiliations:** 1 Department of Orthopedics and Sports Medicine, Erasmus University Medical Centre, Sophia Children’s Hospital, 3015 CN Rotterdam, The Netherlands; 2 Department of Orthopaedic Surgery, Elkerliek Hospital, 5707 HA Helmond, The Netherlands; 3 Department of Surgery, Erasmus University Medical Centre, 3015 CN Rotterdam, The Netherlands; 4 Department of Orthopaedic Surgery, Amphia Hospital, 4818 CK Breda, The Netherlands

**Keywords:** forearm, fracture, pediatric, secondary displacement, angulation, remodeling, pro-supination, diaphyseal, metaphyseal

## Abstract

**Highlights:**

**What are the main findings?**

• Accepted secondary displacements of both metaphyseal and diaphyseal forearm fractures did not lead to worse long-term functional outcomes.
• Predictors of long-term functional impairment included complete initial displacement of the radius, bicortical ulnar fractures, and re-fractures for metaphyseal fractures, as well as older age at trauma for diaphyseal fractures.

**What is the implication of the main finding?**

• A more permissive approach to accepted secondary displacements—particularly for metaphyseal fractures in skeletally immature children—may safely reduce the need for repeat manipulations or surgical interventions.
• Treatment decisions should be tailored by considering patient age, sex, fracture location, and displacement severity.

**Abstract:**

**Background/Objectives:** Conservative management of pediatric forearm fractures remains challenging due to the high incidence of secondary displacement. Given the remarkable remodeling potential of children’s bones, clinicians must decide whether to rely on natural healing or intervene. This study evaluated whether accepted secondary displacements affect long-term outcomes and sought to identify predictors of functional impairment. **Methods:** This retrospective cohort study assessed the long-term outcomes of a cohort of 410 consecutive children who presented with 212 distal metaphyseal and 198 diaphyseal both-bone forearm fractures between 2006–2010. In all patients, closed reduction was recommended for ≥50% displacement, ≥15° angulation (<10 years), or ≥10° angulation (10–16 years). Secondary displacements were frequently accepted, anticipating remodeling. We included 316 children (<16 years) with both-bone forearm fractures (147 diaphyseal, 169 distal metaphyseal), representing 77% of the original cohort, for long-term follow-up (mean 7.2 years, minimum 4 years). Functional and radiographic outcomes were compared between accepted secondary displacements and maintained alignments, stratified by fracture location. Multivariate logistic regression identified predictors of long-term functional impairment, defined as ≥15° loss of pro-supination or QuickDASH ≥ 20. **Results:** In the distal metaphyseal group there were 50 secondary displacements out of 212 fractures, of which 31 were accepted. In the diaphyseal group there were 60 secondary displacements, of which 49 were accepted. At long-term follow-up, patients with accepted secondary displacements had no clinically relevant differences in functional or radiographic outcomes compared with those with maintained alignments across both diaphyseal and distal metaphyseal fracture groups. For distal fractures, complete initial radial displacements, re-fractures, and bicortical ulnar fractures predicted pro-supination loss ≥ 15° or QuickDASH ≥ 20. For diaphyseal fractures, older age at trauma predicted increased risk of pro-supination limitation. **Conclusions:** Accepted secondary displacements did not worsen long-term outcomes, supporting reconsideration of strict reduction criteria. The substantial remodeling capacity of pediatric bone—especially in distal metaphyseal fractures in skeletally immature children—should be emphasized when making treatment decisions to avoid unnecessary surgical interventions.

## 1. Introduction

Despite the well-recognized remodeling potential of angulated pediatric forearm fractures, they are still considered unpredictable and the best treatment strategy remains uncertain [1]. Clinicians are often faced with the difficult decision of whether to let a displaced fracture heal through natural remodeling or to perform a reduction. This decision is frequently based on gut feeling rather than objective criteria.

Do et al. highlighted this dilemma, stating, “Despite the remarkable potential for remodeling seen in pediatric forearm fractures, there is still a natural tendency to try to make each fracture radiographically more anatomic” [2]. Management of pediatric forearm fractures ranges from cast immobilization alone to fracture reduction with or without supplementary stabilization, yet secondary displacement has been reported in as many as 46% of diaphyseal both-bone fractures [3] and 51% of initially displaced metaphyseal forearm fractures and when treated without stabilization [4]. Non-anatomical healing can lead to malunion, which may subsequently cause restricted forearm rotation and noticeable deformity [5]. However, the pediatric skeleton has a remarkable capacity for remodeling, correcting angular malunions over time [6]. This process is governed by the Hueter-Volkmann and Wolff’s laws, with remodeling potential influenced by patient age, fracture proximity to the physis, and the plane of deformity [7]. Mid-shaft fractures are known to remodel less reliably and may leave residual angular deformity and restricted rotation [8]. Angulated distal forearm fractures in particular remodel well, with rates of up to 2.4° per month reported [9]. Consequently, conservative treatment of angulated distal forearm fractures in children often yields satisfactory long-term outcomes [10]. Due to this remodeling potential, certain degrees of forearm fracture (re)displacement can be safely accepted in the expectation that remodeling will occur. Orland et al. suggested that one-quarter of closed reductions of distal forearm fractures in children under ten years may be unnecessary [11].

Nevertheless, an increasing tendency toward operative treatment of pediatric forearm fractures has been reported [12]. Modern parents are increasingly well-informed and often expect an optimal radiographic and clinical outcome for their child. This can lead to pressure for more aggressive treatment, including operative intervention, to achieve near-anatomic alignment [13]. Long-term studies, however, have not shown superior outcomes for surgical intervention. Eismann et al. stated “Clinical research fails to support more aggressive management of pediatric upper extremity fractures” [14]. Recent literature echoes these concerns: Franklin et al. strongly emphasized that high-level evidence to support clinical decision-making remains limited and explicitly called for well-designed studies with long-term follow-up, using forearm pro-supination as the primary outcome measure [15]. Addressing this gap, we investigated long-term outcomes in a large cohort of children with both-bone forearm fractures. The aims of this study were (1) to determine whether accepted secondary displacements affect long-term functional outcomes, and (2) to identify predictors of long-term functional impairment.

## 2. Materials and Methods

### 2.1. Study Design, Setting, and Participants

This study reports the long-term outcomes of a cohort of all consecutive children who presented at the emergency department with both-bone forearm fractures between 2006 and 2010 and were originally enrolled in a series of previously published randomized controlled trials (RCTs) [16,17,18,19,20]. The original cohort had a mean follow-up of seven months. For the present analysis, patients were re-evaluated after a minimum follow-up of four years. Children under 16 years of age with diaphyseal or metaphyseal both-bone forearm fractures were eligible for inclusion. Metaphyseal forearm fractures were defined according to the AO Pediatric Comprehensive Classification of Long-Bone Fractures. The metaphysis was determined by a square with side lengths equal to the widest part of the distal growth plate; in paired bones such as the radius and ulna, both bones were included within this square. Fractures with their center located within this square were classified as metaphyseal (AO 23-M). Fractures located proximal to this region and involving the tubular shaft were classified as diaphyseal (AO 22-D) [21]. Open fractures were excluded.

This study was conducted and reported in accordance with the STROBE (Strengthening the Reporting of Observational Studies in Epidemiology) guidelines for observational cohort studies.

### 2.2. Interventions

Closed reduction was indicated for fractures demonstrating ≥ 50% displacement, angulation ≥ 15° in children younger than 10 years, or angulation ≥ 10° in children aged 10–16 years, irrespective of whether the angulation occurred in the sagittal or coronal plane. Secondary displacement was defined as recurrent displacement meeting these criteria during cast immobilization, for which re-manipulation was advised in all cases.

However, surgeons were often reluctant to perform a repeat intervention and accepted the secondary displacement, anticipating remodeling. Untreated secondary displacements were classified as accepted secondary displacements, which consolidated in a malunited position. Alignments not meeting secondary displacement criteria were considered maintained alignments.

Participants were managed according to predefined treatment algorithms derived from previously published randomized controlled trials, with treatment allocation determined by fracture location, need for reduction, and fracture stability, as outlined below. Metaphyseal fractures not requiring reduction were randomized to treatment with either a below-elbow cast (BEC) or an above-elbow cast (AEC) for four weeks, with the wrist positioned in neutral [16]. Metaphyseal fractures requiring reduction underwent closed reduction under general anesthesia, followed by a fluoroscopic stress test to assess stability. Fractures that re-displaced during maximum passive pronation or supination were classified as unstable and treated with K-wire fixation, whereas stable reduced fractures were randomized to treatment with or without K-wire fixation [17]. The K-wires were placed transcutaneously to facilitate removal in the outpatient clinic. Diaphyseal fractures that were non-reduced or stable after reduction were randomized to receive an AEC for six weeks or early conversion to a BEC after three weeks (AEC- > BEC) [18,19]. Unstable diaphyseal fractures underwent stabilization with one or two intramedullary nails [20].

### 2.3. Outcome Measures

The primary outcome measure was long-term functional impairment, defined as a forearm pro-supination deficit of ≥15° or a QuickDASH score ≥ 20 points. Data are presented as mean ± SD. Scores on the QuickDASH range from 0 (no disability) to 100 (maximum disability). Previously, a 20-point change was identified as the minimal clinically important difference (MCID) for the QuickDASH [22]. Forearm pro-supination was measured using a 180° goniometer made of transparent, flexible plastic with 30 cm arms. Forearm pronation and supination were measured bilaterally. Loss of forearm pro-supination was defined as the difference between the total arc of motion (pronation + supination) of the affected forearm and that of the contralateral, unaffected forearm. Long-term functional impairment was defined using the minimum forearm pronation (65°) and supination (77°) required for the performance of daily activities [23]. Based on these functional thresholds, a forearm pronation–supination deficit of ≥15° was considered indicative of long-term functional impairment following a both-bone forearm fracture. The secondary outcomes included radiographic assessment at final follow-up, measuring coronal and sagittal angulation of both forearm bones; the JAMAR grip strength ratio, defined as the grip strength of the injured wrist relative to the contralateral side; the ABILHAND-kids questionnaire [24]; and Numeric Rating Scale (NRS) scores for cosmetic appearance.

Clinical outcomes were analyzed by comparing patients with accepted secondary displacement and those with maintained alignment at fracture consolidation. Subgroup analyses were performed for metaphyseal and diaphyseal fractures. Additionally, potential predictors of long-term functional impairment were analyzed, including age at injury, anatomical fracture location, fracture configuration (greenstick versus bicortical), complete initial displacement of the radius or ulna, secondary displacement, and occurrence of re-fracture. Re-fracture was defined as a new fracture through the consolidated fracture site occurring after both clinical and radiographic healing had been achieved.

### 2.4. Data Collection and Bias Control

To minimize observer bias, short-term follow-up examinations were performed by one orthopedic surgeon, whereas a different orthopedic surgeon performed the long-term follow-up evaluations, blinded to allocation.

**Figure 1:**
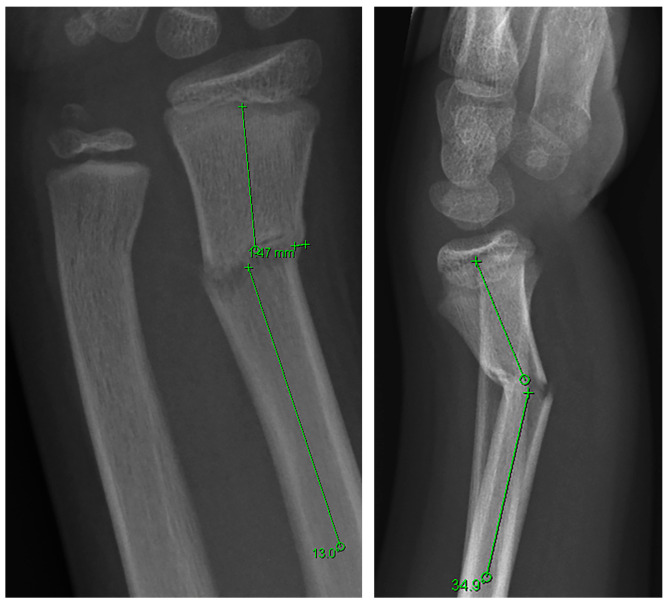
Radiographic measurement technique: Pre-reduction posteroanterior (PA) and lateral radiographs of an 11-year-old boy with a distal metaphyseal forearm fracture. The PA view demonstrates 13.0° of radial angulation of the radius, and the lateral view demonstrates 34.9° of dorsal angulation.

Radiographic angulation was assessed using a standardized measurement technique previously described and illustrated in Figure 1 [3]. Angulation was defined as the angular deviation between the longitudinal axes of the proximal and distal fracture fragments. All measurements were performed in a blinded manner by one of the coauthors (PE). To evaluate inter-rater reproducibility, two authors (LD and PE) independently measured the radiological angulations of 45 patients, blinded to allocation. Interrater reliability yielded intraclass correlation coefficients (ICCs) of 0.9 (95% CI, 0.8–0.9) for radial angulation measured in the coronal and sagittal planes, and ICCs of 0.8 (95% CI, 0.7–0.9) and 0.9 (95% CI, 0.9–1.0) for ulnar angulation in the coronal and sagittal planes, respectively.

### 2.5. Statistical Analysis

Patients lost to follow-up were compared with those included in the study with regard to demographic characteristics. Categorical variables were analyzed using the chi-square test, whereas continuous variables were compared using independent-samples t tests. Radiographic and functional outcomes were compared between patients with accepted secondary displacement and those with maintained alignment using independent-samples t tests. Univariate logistic regression analyses were performed to identify predictors of long-term functional impairment, and variables with a *p* value < 0.10 were entered into a multivariable logistic regression model. Statistical significance was defined as *p* < 0.05. All statistical analyses were performed using IBM SPSS Statistics for Windows, version 29.0 (IBM Corp., Armonk, NY, USA).

## 3. Results

### 3.1. Participants, Fracture Characteristics and Treatment

Between 2014 and 2016, long-term follow-up data were available for 316 of 410 eligible participants (77%), with a mean follow-up duration of 7.2 years (range, 4.2–10.3 years). The study population included 147 diaphyseal fractures (46%) and 169 distal metaphyseal fractures (54%), and the mean age at the time of injury was 8.0 years (±3.3 years). No significant differences in demographic characteristics were found between patients included in the analysis and those lost to follow-up (Table 1).

#### 3.1.1. Distal Metaphyseal Fractures

**Figure 2:**
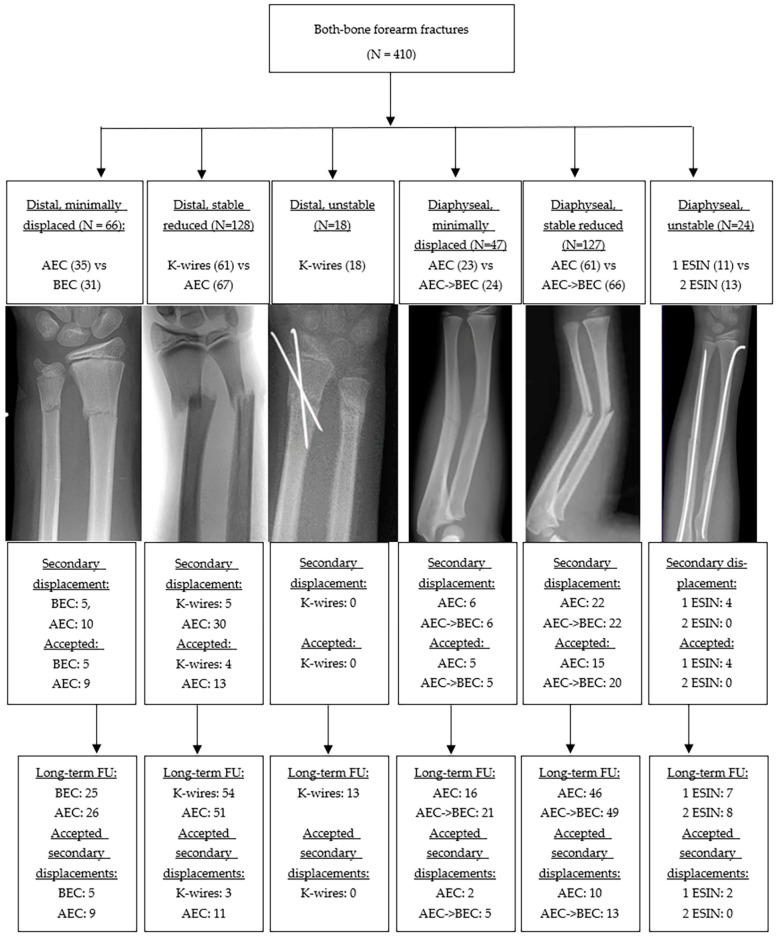
Inclusion Flowchart.

An inclusion flowchart is presented in Figure 2. Of the 212 fractures in this group, 66 (31%) were minimally displaced and treated conservatively, while 146 (69%) were displaced and underwent closed reduction. Among the reduced fractures, 128 (92%) were classified as stable and randomized to either K-wire fixation or cast treatment, while 18 (8%) were deemed unstable and were therefore managed with K-wire fixation. In patients treated without stabilization, secondary displacement was observed in 30 of 67 (45%) reduced metaphyseal fractures and in 15 of 66 (23%) minimally displaced fractures managed in a cast. Secondary displacement was accepted in 31 of 50 cases (62%). Patients with accepted secondary displacement were significantly younger at the time of trauma than those with maintained alignment (7.2 years vs. 8.6 years, *p* = 0.03), with no significant difference in sex distribution between groups (68% vs. 62% male; *p* = 0.57).

Excluding secondary displacement, minor complications occurred in 22 of 78 patients (28%) who underwent K-wire fixation, compared with 6 of 127 patients (4.7%) treated without fixation. Reported complications consisted of transient superficial radial nerve neuropraxia, subcutaneous K-wires necessitating surgical removal, wound dehiscence, and superficial infections that resolved without further operative intervention. Re-fractures occurred in 11% of patients.

#### 3.1.2. Diaphyseal Fractures

Of the diaphyseal fractures, 47 of 198 (24%) were minimally displaced, whereas 151 (76%) were displaced and managed with closed reduction. Of the reduced fractures, 127 (84%) were stable and 24 (16%) were unstable, requiring intramedullary nailing. Among patients who were managed without stabilization, secondary displacement was observed in 44 of 127 reduced fractures (35%) and in 12 of 47 minimally displaced fractures (26%). Secondary displacement was accepted in 45 of 56 cases (80%). No statistically significant differences were observed in age at trauma (7.8 vs. 7.7 years, *p* = 0.90) or in gender distribution (60% vs. 67% male, *p* = 0.39) between patients with accepted secondary displacement and those who maintained alignment. Minor complications were observed in 9 of 24 patients (38%) who underwent intramedullary nailing, compared with 20 of 174 patients (11%) managed without nailing. Re-fractures were seen in 24 of 149 diaphyseal fractures (16%).

### 3.2. Radiographic & Functional Outcomes

#### 3.2.1. Outcomes After Distal Metaphyseal Fractures

Although patients with accepted secondary displacements of distal metaphyseal fractures had statistically significantly inferior radiographic and functional outcomes at short-term follow-up, no statistically significant differences in long-term radiographic or functional outcomes were observed compared with fractures that maintained alignment (see Table 2A,B). At final follow-up, 12% of patients had a pro-supination limitation of ≥15° (11% in the maintained-alignment group vs. 18% in the accepted secondary displacement group, *p* = 0.34). Furthermore, 6.2% of patients had a QuickDASH score ≥ 20 (5.8% in the maintained-alignment group and 8.7% in the accepted secondary displacement group, *p* = 0.59).

#### 3.2.2. Outcomes After Diaphyseal Fractures

Similarly, patients with accepted secondary displacements of diaphyseal fractures had significantly worse radiographic and functional outcomes at short-term follow-up, but no clinically relevant differences in long-term outcomes were observed when compared with fractures that maintained alignment (see Table 2C,D). Radiographically, a significant difference was observed only in sagittal angulation of the radius (5.6° vs. 3.8°). Functionally, there were no differences in long-term outcomes. At final follow-up, 20% of patients with diaphyseal fractures had a pro-supination limitation of ≥15° (20% in the maintained-alignment group vs. 22% in the accepted secondary displacement group, *p* = 0.82). Additionally, 7.8% of patients had a QuickDASH score ≥ 20 (9.1% in the maintained-alignment group vs. 3.1% in the accepted secondary displacement group, *p* = 0.27).

### 3.3. Predictors of Long-Term Functional Impairment

Results from multivariate analyses are presented in Table 3A,B. For distal forearm fractures, complete initial displacement of the radius and the occurrence of re-fracture were associated with a ≥15° limitation in forearm pro-supination at long-term follow-up, while a bicortical ulnar fracture predicted a QuickDASH score ≥ 20. For diaphyseal fractures, older age at the time of injury was associated with a higher risk of a ≥15° limitation in forearm pro-supination at long-term follow-up. Patients with complete diaphyseal fractures of both the radius and ulna demonstrated a significantly greater loss of forearm pro-supination at long-term follow-up compared with patients with at least one incomplete fracture (mean limitation 8.4° vs. 4.2°, *p* = 0.04). However, in uni- and multivariable logistic regression analyses assessing factors associated with a clinically relevant limitation of ≥15° of forearm rotation, a complete both-bone diaphyseal forearm fracture was not independently associated with this outcome (*p* = 0.11).

## 4. Discussion

This study aimed to address two key questions: (1) whether accepted secondary displacements result in poorer long-term outcomes in children with metaphyseal and diaphyseal forearm fractures; (2) which factors contribute to functional impairment at long-term follow-up in children with both-bone forearm fractures.

### 4.1. Metaphyseal Forearm Fractures

Our findings confirm that accepted secondary displacements after distal metaphyseal fractures did not compromise long-term outcomes compared with fractures that maintained alignment. Although patients who had an accepted secondary displacement were significantly younger at the time of trauma than those who did not (mean age of 7.2 years vs. 8.6 years, *p* = 0.03), both ages have similar remodeling capacity with at least 5 years of remaining growth. These young patients with secondary displacements exceeding 15° who did not undergo further intervention still achieved long-term outcomes comparable to those who maintained acceptable alignments. The observed outcomes indicate that the radiographic criteria we applied may have been too strict—particularly for the younger patients, in whom remodeling capacity is greatest.

Previously, in a cohort of 232 pediatric distal forearm fractures, Zimmermann et al. reported that substantial displacement (>20° angulation) in children younger than 10 years had no adverse effect on long-term results [10]. Similarly, Crawford et al. accepted distal radius fractures with complete (100%) dorsal translation in 51 children under 10 years of age and observed excellent outcomes in all cases [6]. In a series by Hove et al., eight patients with more than 15° of malangulation at union were re-examined after 7 years, at which time all fractures had completely remodeled and demonstrated normal function [25]. With respect to remodeling, Jeroense et al. observed an average remodeling rate of approximately 2.4° per month in children younger than 14 years with distal radius fractures demonstrating sagittal angulations of at least 15° [9]. Likewise, Lynch et al. described comparable remodeling rates of about 2.3° per month in the coronal plane [26]. Thus, remodeling capacity in the coronal plane may be greater than previously assumed.

Numerous recommendations have been proposed regarding the degree of angulation that can be safely accepted in distal metaphyseal forearm fractures. In 2005, Wilkins and O’Brien reported that dorsal angulations of up to 30–35° may remodel satisfactorily in children with at least five years of remaining growth [27]. More recently, van Delft et al. recommended accepting up to 30° of dorsal angulation and 15–20° of radial angulation in children ≤ 9 years of age, and up to 20° dorsal and 10° radial angulation in older children [28]. In addition to chronological age, remodeling capacity is influenced by biological sex. Because physeal closure occurs earlier in girls (mean age 12.9 years) than in boys (mean age 14.5 years), boys retain a longer growth period and, consequently, demonstrate greater remodeling capacity at comparable ages [29].

**Figure 3:**
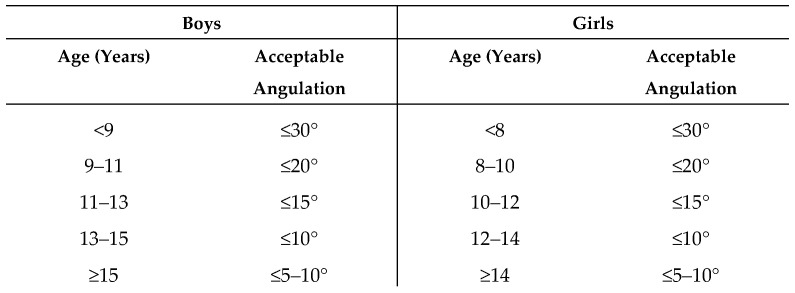
Acceptable Angulation for Distal Metaphyseal Forearm Fractures.

Based on our findings, we interpreted the absence of inferior long-term outcomes in children with accepted secondary displacement as evidence that residual angulation—within certain limits—can be safely tolerated in patients with sufficient remaining growth. Accordingly, rather than deriving strict angulation cut-off values from statistical thresholds within our cohort, we developed clinically applicable recommendations by integrating our outcome data with the above-mentioned established treatment guidelines and remodeling data from the literature, while explicitly accounting for sex-related differences in skeletal maturation. Our age- and sex-specific recommendations for acceptable residual sagittal angulation, summarized in Figure 3, are intended to reflect expected remodeling capacity across pediatric age groups and to support informed clinical decision-making.

These individualized recommendations underscore the importance of considering both age and sex when determining whether reduction is necessary, and they suggest that many mild deformities may be safely accepted without compromising long-term outcomes. Surprisingly, treatment recommendations also vary by provider, as hand surgeons are approximately three times more likely than pediatric orthopedic surgeons to recommend operative management for the same child with a distal radius fracture [30]. Future randomized controlled trials, such as AFIC [31] and CRAFFT [32], will clarify the necessity of reduction in younger children with severely displaced fractures.

Furthermore, not only the degree of angulation, but also the pattern of displacement appears to be clinically relevant when assessing the risk of secondary displacement. Zamzam et al. identified complete initial radial displacement and both-bone fractures as strong predictors of secondary displacement, with odds ratios of 25 and 23, respectively [33]. Similarly, van Delft et al. evaluated 200 pediatric patients with displaced distal forearm fractures and observed that although most (83%) closed reductions performed in the emergency department were successful, the cases taken to the operating theatre—likely representing more severe injury patterns—showed a high rate of secondary displacement (47%) when no additional K-wire fixation was used. In that study, complete initial displacement was also strongly associated with unsuccessful reduction (86%) [28]. Our findings align with these observations: complete displacement of the radius, bicortical involvement of the ulna, and re-fractures indicate a more severe injury pattern and correlate with both a higher risk of secondary displacement and increased long-term functional impairment.

### 4.2. Diaphyseal Forearm Fractures

Long-term functional limitations were more pronounced when the fracture involved the diaphysis rather than the distal metaphysis, reflecting the inferior remodeling potential at mid-shaft level. Although accepted secondary displacement in diaphyseal fractures resulted in slightly greater residual sagittal angulation (5.6° vs. 3.8° in patients who maintained alignment), this difference did not translate into worse functional outcomes. Nonetheless, diaphyseal fractures resulted in greater long-term pro-supination loss than metaphyseal fractures. This finding aligns with Gandhi et al., who emphasized that mid-shaft deformities remodel poorly [34]. Kay et al. reported that both-bone midshaft forearm fractures in children older than 10 years more frequently result in residual functional deficits than is commonly appreciated, and concluded that closed treatment resulting in more than 10° of malalignment should therefore not be accepted in this age group [8].

**Figure 4:**
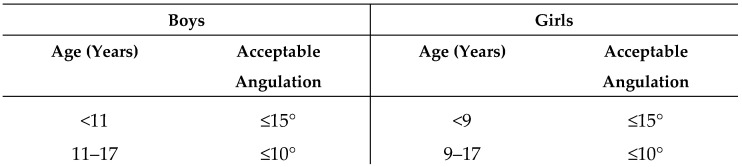
Acceptable Angulation for Diaphyseal Forearm Fractures (Bowman).

Price et al. found complete remodeling in only a minority of severe diaphyseal malunions, mostly in children under 10 years [35]. Zionts et al. demonstrated that residual angulations < 10° resulted in excellent outcomes in 75% of cases, whereas outcomes were excellent in only 40% of cases with residual angulations > 15° [5]. Ploegmakers et al. confirmed that acceptable angulation thresholds decrease as age increases [1]. These findings reinforce the need for individualized management strategies, particularly for children older than 10 years. Bowman et al. proposed the following treatment recommendations for diaphyseal forearm fractures in children, provided in Figure 4 [3]. Our study agrees with the criteria proposed by Bowman, as diaphyseal injuries exhibited greater residual limitations in forearm rotation at long-term follow-up than distal fractures. Therefore, we would not advise accepting greater angulation in these injuries, particularly given their lower remodeling potential compared with metaphyseal fractures.

### 4.3. Factors Associated with Functional Impairment

Factors associated with poorer long-term motion included older age at injury, diaphyseal involvement, bicortical ulnar fractures, complete radial displacement, and the occurrence of re-fractures. Complete displacement of the radius and bicortical ulnar fractures both suggest a higher-energy mechanism and greater soft-tissue damage. Diaphyseal fracture location and a higher age at the time of injury were both associated with significantly greater pro-supination loss at follow-up, consistent with their reduced remodeling potential. Re-fractures contributed to worse outcomes, likely due to repeated injury to the inter-osseous membrane and the need for prolonged immobilization.

### 4.4. Limitations

This study has several limitations. The relatively high rate of secondary displacement observed after conservative treatment in our cohort may be explained by the exclusive inclusion of both-bone forearm fractures, which are inherently less stable than isolated distal radius fractures and therefore more prone to redisplacement. In the present study, closed reduction was performed in all fractures with ≥50% displacement according to the treatment protocol. However, existing literature suggests that greater degrees of displacement may be safely accepted in younger children with substantial remaining remodeling potential [6]. Surgical indications reflected local institutional protocols. In retrospect, our results suggest that thresholds for both reduction and surgical fixation may have been relatively low, as long-term outcomes remained excellent even when secondary displacement was accepted without further intervention. It should be acknowledged that in many pediatric trauma centers, comparable fractures may have been managed non-operatively. This variability in practice highlights the absence of universally accepted surgical thresholds. Decisions regarding re-manipulation were shared between surgeons, parents, and children, introducing possible selection bias. Younger children, who have greater remodeling potential, were more likely to have secondary displacements accepted. Accordingly, patients with accepted secondary displacement were significantly younger at the time of trauma compared with those who maintained alignment (7.2 vs. 8.6 years, *p* = 0.03). Reduction criteria were not tailored according to fracture location or sex, and age was dichotomized into <10 or ≥10 years, which may have been overly broad and did not account for sex-related differences in remodeling potential. Range of motion was assessed by a single orthopedic surgeon, potentially introducing measurement bias.

**Figure 5:**
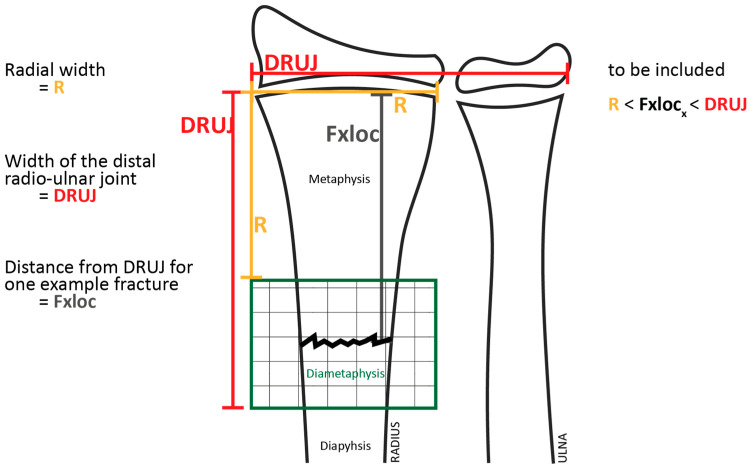
Definition of diametaphyseal transition zone. Reproduced from Wollkopf et al. [36], with permission from the authors and the publisher.

Furthermore, although fractures were classified according to the AO Pediatric Comprehensive Classification of Long-Bone Fractures, this study did not analyze diametaphyseal forearm fractures as a separate subgroup. According to the AO definition, the metaphysis is determined by a square that includes both bones in paired structures such as the radius and ulna; therefore, in a cohort consisting exclusively of both-bone forearm fractures, the region that is sometimes described as the diametaphyseal junction falls within the metaphyseal region [36]. Nevertheless, we acknowledge that fractures at the diametaphyseal junction (Figure 5) likely differ biologically from purely metaphyseal and midshaft diaphyseal fractures, with remodeling potential expected to be lower than in metaphyseal fractures and higher than in diaphyseal fractures. Future studies should therefore specifically investigate acceptable angulation thresholds and remodeling behavior for diametaphyseal forearm fractures as a distinct entity.

## 5. Conclusions

Accepted secondary displacement did not result in inferior long-term outcomes in either metaphyseal or diaphyseal fractures, suggesting that conventional reduction criteria may be too strict. Complete initial displacement, re-fracture, and a bicortical ulnar fracture were predictors of functional impairment in distal fractures, while older age at trauma predicted impairment in diaphyseal fractures. These results emphasize the exceptional remodeling capacity of pediatric bone—especially in the distal metaphysis—and support patient-tailored treatment approaches that avoid unnecessary surgical interventions.

## Figures and Tables

**Table 1:** Representation of Follow-Up Population.

	Included for Long-Term FU (N = 316)	Lost to FU (N = 94)	Mean Difference (95% CI)	*p*-Value
Age at trauma	8.0 (±3.3)	8.4 (±3.6)	−0.4 (−1.2 to 0.4)	0.29
Male sex	60% (191)	70% (66)	−9.8% (−21 to 1)	0.09
Bicortical Radius Fracture	53% (167)	56% (53)	−3.2% (−15 to 9)	0.59
Bicortical Ulnar Fracture	39% (122)	40% (37)	−1.0% (−13 to 10)	0.86
Secondary displacement rate	27% (84)	27% (26)	0.0% (−10 to 10)	0.99
Accepted Secondary displacements	19% (59)	21% (11)	−2.6% (−12 to 7)	0.57
Loss in Pro-Supination at 6 m FU	11.6° (±13.8)	13.9° (±15.1)	−2.3° (−6 to 1)	0.17
Complications	27% (86)	29% (27)	−1.2% (−12 to 9)	0.82

**Table 2:** (**A**) Radiographic outcomes after distal metaphyseal fractures, (**B**) Functional outcomes after distal metaphyseal fractures, (**C**) Radiographic outcomes after diaphyseal fractures and (**D**) Functional outcomes after diaphyseal fractures.

	Accepted Secondary Displacement	Maintained Alignment	*p*-Value
(**A**)			
**Distal** **metaphyseal fractures**			
**Consolidation (T = 6 weeks)**			
Radius—PA	8.0° (±7°)	5.1° (±5°)	0.004
Radius—Lateral	19.0° (±9°)	8.4° (±8°)	<0.001
Ulna—PA	7.0° (±4°)	5.5° (±4°)	0.049
Ulna—Lateral	6.3° (±5°)	5.1° (±4°)	0.20
**Short-term FU (T = 6 months)**			
Radius—PA	5.5° (±5°)	3.0° (±3°)	<0.001
Radius—Lateral	9.5° (±5°)	5.0° (±4°)	<0.001
Ulna—PA	4.8° (±4°)	4.0° (±3°)	0.17
Ulna—Lateral	4.5° (±3°)	3.2° (±3°)	0.044
**Long-term FU (T ≥ 4 years)**			
Radius—PA	4.9° (±3°)	4.9° (±4°)	0.99
Radius—Lateral	4.2° (±3°)	3.8° (±3°)	0.57
Ulna—PA	5.0° (±3°)	4.9° (±3°)	0.79
Ulna—Lateral	3.1° (±3°)	3.5° (±3°)	0.60
(**B**)			
**Short-term FU (6 months)**			
Loss of pro-supination	16.5° (±17°)	7.8° (±9°)	<0.001
NRS cosmetics (parents)	8.6 (±2)	8.6 (±2)	0.84
NRS cosmetics (surgeon)	9.0 (±1)	9.0 (±1)	0.75
ABILHAND questionnaire	41.7 (±1)	41.2 (±4)	0.53
**Long-term FU (≥4 years)**			
Loss of pro-supination	4.5° (±9°)	4.3° (±7°)	0.87
NRS cosmetics (parents)	9.1 (±1)	8.7 (±2)	0.17
NRS cosmetics (surgeon)	9.8 (±1)	9.6 (±1)	0.14
ABILHAND questionnaire	41.4 (±1)	41.6 (±1)	0.54
QuickDASH questionnaire	4.7 (±9)	4.0 (±8)	0.66
JAMAR grip strength	100%	99%	0.66
(**C**)			
**Diaphyseal fractures**			
**Consolidation (T = 6 weeks)**			
Radius—PA	7.5° (±5°)	5.8° (±5°)	0.73
Radius—Lateral	13.1° (±6°)	8.7° (±6°)	<0.001
Ulna—PA	5.6° (±4°)	5.0° (±4°)	0.84
Ulna—Lateral	7.5° (±5°)	5.4° (±4°)	0.26
**Short-term FU (T = 6 months)**			
Radius—PA	6.9 (±5°)	5.8° (±4°)	0.11
Radius—Lateral	10.2° (±5°)	7.4° (±5°)	<0.001
Ulna—PA	6.3° (±4°)	5.0° (±4°)	0.045
Ulna—Lateral	6.7° (±5°)	4.5° (±4°)	0.002
**Long-term FU (T ≥ 4 years)**			
Radius—PA	9.1° (±3°)	9.2° (±3°)	0.86
Radius—Lateral	5.6° (±4°)	3.8° (±3°)	0.007
Ulna—PA	5.3° (±3°)	5.2° (±3°)	0.88
Ulna—Lateral	4.7° (±3°)	4.5° (±3°)	0.70
(**D**)			
**Short-term FU (6 months)**			
Loss of pro-supination	21.6° (±17°)	13.2° (±15°)	0.001
NRS cosmetics (parents)	7.2 (±2)	8.3 (±2)	0.002
NRS cosmetics (surgeon)	7.6 (±2)	8.4 (±2)	0.01
ABILHAND questionnaire	39.4 (±9)	40.9 (±6)	0.19
**Long-term FU (≥4 years)**			
Loss of pro-supination	6.7° (±9°)	7.5° (±15°)	0.78
NRS cosmetics (parents)	8.0 (±2)	8.4 (±2)	0.35
NRS cosmetics (surgeon)	9.6 (±1)	9.5 (±1)	0.69
ABILHAND questionnaire	41.8 (±1)	40.5 (±5)	0.19
QuickDASH questionnaire	3.6 (±5)	5.5 (±10)	0.31
JAMAR grip strength	94%	98%	0.27

**Table 3:** (**A**) Multi-variate Linear regression analysis—Distal fractures. (**B**) Multi-variate Linear regression analysis—Diaphyseal fractures.

	Models			
	≥15° Loss in Pro-Sup		QuickDASH ≥ 20 Points	
	Exp (B)	Significance	Exp (B)	Significance
(**A**)				
Complete Initial Displacement Radius	6.1	0.007	-	-
Re-Fracture	4.8	0.02	-	-
Bicortical Ulna Fracture	-	-	5.7	0.014
(**B**)				
Age at Trauma	1.2	0.02	-	-

## Data Availability

The data presented in this study are available on request from the corresponding author due to ethical and privacy restrictions.

## Abbreviations

The following abbreviations are used in this manuscript:

BEC	Below elbow cast
AEC	Above elbow cast
K-wire	Kirschner wire
ESIN	Elastic stable intramedullary nail
RCT(s)	Randomized Controlled Trial(s)
QuickDASH	Quick Disabilities of the Arm, Shoulder and Hand questionnaire
SD	Standard Deviation
SDD	Smallest detectable difference
NRS	Numeric rating scale
ICC	Intraclass Correlation
CI	Confidence Index
FU	Follow-up
IBM SPSS	International Business Machines—Statistical Package for the Social Sciences
AFIC	Angulated fractures of the distal forearm in children
CRAFFT	Children’s Radius Acute Fracture Fixation Trial
JAMAR	JAMAR Hydraulic Hand Dynamometer
